# miR-31 from Mesenchymal Stem Cell-Derived Extracellular Vesicles Alleviates Intervertebral Disc Degeneration by Inhibiting NFAT5 and Upregulating the Wnt/*β*-Catenin Pathway

**DOI:** 10.1155/2022/2164057

**Published:** 2022-10-20

**Authors:** Baodong Wang, Na Xu, Li Cao, Xiaojun Yu, Shanxi Wang, Qikun Liu, Yinguang Wang, Haoran Xu, Yang Cao

**Affiliations:** ^1^Department of Orthopedics, The First Affiliated Hospital of Harbin Medical University, Harbin 150001, China; ^2^Prenatal Diagnosis Center, The Sixth Affiliated Hospital of Harbin Medical University, Harbin 150001, China; ^3^Student Affairs Office, Heilongjiang Nursing College, Harbin 150000, China; ^4^Department of Orthopedics, Tongji Hospital, Tongji Medical College, Huazhong University of Science and Technology, Wuhan 430030, China

## Abstract

In this study, we explored the regulatory mechanism of intervertebral disc degeneration (IDD) that involves miR-31 shuttled by bone marrow mesenchymal stem cell-derived extracellular vesicles (BMSC-EVs) and its downstream signaling molecules. Nucleus pulposus cells (NPCs) were isolated and treated with TNF-*α* to simulate IDD *in vitro*. The TNF-*α*-exposed NPCs were then cocultured with hBMSCs or hBMSC-EVs *in vitro* to detect the effects of hBMSC-EVs on NPC viability, apoptosis, and ECM degradation. Binding between miR-31 and NFAT5 was determined. A mouse model of IDD was prepared by vertebral disc puncture and injected with EVs from hBMSCs with miR-31 knockdown to discern the function of miR-31 *in vivo*. The results demonstrated that hBMSC-EVs delivered miR-31 into NPCs. hBMSC-EVs enhanced NPC proliferation and suppressed cell apoptosis and ECM degradation, which was associated with the transfer of miR-31 into NPCs. In NPCs, miR-31 bound to the 3′UTR of NFAT5 and inhibited NFAT5 expression, leading to activation of the Wnt/*β*-catenin pathway and thus promoting NPC proliferation and reducing cell apoptosis and ECM degradation. In addition, miR-31 in hBMSC-EVs alleviated the IDD in mouse models. Taken together, miR-31 in hBMSC-EVs can alleviate IDD by targeting NFAT5 and activating the Wnt/*β*-catenin pathway.

## 1. Introduction

Low back pain is common and associated with intervertebral disc degeneration (IDD) [1, 2]. Back pain bothers most people, reportedly to be around 80% of all adults worldwide [3]. The incidence of lower back pain increases with age, and back pain is the number one cause of disability in the elderly [4]. In addition to the elderly, back pain also significantly limits the activity of younger adults [3]. Additionally, low back pain is characterized as the number one cause of disability globally back, showing increasing health and financial burden [5]. Therefore, it is essential to discern the mechanism of IDD that leads to back pain and find better treatments.

The pathogenesis of IDD remains to be completely understood. It is believed that increased degradative enzymes and proinflammatory cytokines as well as reductions in matrix proteins are major mechanisms for the progression of IDD [6]. There are two major components in intervertebral disc (IVD): annulus fibrosus and nucleus pulposus (NP), and the latter is centrally located in the IVD and therefore important in the function and structure of IVD [2]. Importantly, NP cell (NPC) apoptosis has been shown to be involved in IDD [7]. Here, we focused on studying apoptosis, inflammatory response, and the expression of extracellular matrix- (ECM-) related genes in NPCs.

Bone marrow mesenchymal stem cell-derived extracellular vesicles (BMSC-EVs) have been documented to inhibit IDD [8]. EVs carry many molecules, including proteins, lipids, and RNAs involved in cell communications [9]. MicroRNA- (miR-) 31 has been reported to be carried by MSC-EVs [10]. miR-31 is well known for regulating cell functions, including cell apoptosis and proliferation [11, 12]. A recent study has identified miR-31 as a key regulator of IDD since its overexpression facilitates NPC proliferation and ECM formation, inhibits NPC apoptosis, and reduces the level of matrix-degrading enzymes in NPCs [13]. Importantly, miR-31 has been identified to target NFAT5 and regulate its expression in Ewing Sarcoma [14]. NFAT5, one of the members of the NFAT family, is a transcription factor that regulates cell functions, including cell invasion and apoptosis [15]. NFAT5 bears great responsibility in the postnatal homeostasis of the spine and controls a variety of functions, including cellular osmoadaptation and axial skeleton embryogenesis [16]. Importantly, NFAT5 is involved in the inflammatory response and osmotic loading in NPCs [17]. Moreover, previous data have shown that NFAT5 inhibits the activation of the Wnt pathway [18]. Wnt/*β*-catenin is highly conserved during evolution and closely associated with inflammation [19]. In addition, activation of the Wnt/*β*-catenin pathway possesses potentials to modulate cell behavior, cell fate, cell proliferation, and survival in both embryos and adults [20]. Importantly, the Wnt pathway has been shown to be involved in the progression of IDD through inflammation-related mechanisms [21]. Notably, previous research has shown that miR-31 can regulate tumorigenesis through activation of the Wnt signaling pathway [22]. Therefore, in this study, we aimed to investigate whether bone marrow mesenchymal stem cell-derived EVs (BMSC-EVs) carried miR-31 to be involved in IDD through regulation of the NFAT5/Wnt/*β*-catenin axis.

## 2. Materials and Methods

### 2.1. Ethics Statement

Isolation experiments of NPCs were approved by the Ethics Committee of Tongji Hospital, Tongji Medical College, Huazhong University of Science and Technology. The study involving humans was ratified by the Medical Ethics Committee of Tongji Hospital, Tongji Medical College, Huazhong University of Science and Technology, and was consistent with the *Declaration of Helsinki*. An informed consent form was signed by each participant. Animal experiments were completed under the ratification of the Animal Ethics Committee of Tongji Hospital, Tongji Medical College, Huazhong University of Science and Technology.

### 2.2. Microarray-Based Analysis

Downstream genes of miR-31 were predicted utilizing RAID (score > 0.95), TargetScan (cumulative weighted context + +score≦−0.5), mirDIP (integrated score > 0.7), and miRWalk databases (energy < −20, accessibility < 0.01, au > 0.55). Then, the Venn diagram was plotted to obtain the key genes using the jvenn tool [23]. A PPI network of the downstream genes and intersected key genes was constructed using GeneMANIA [24]. According to the map plotted using Cytoscape [25], the core degree was determined, and the genes with the highest core degree were selected to further predict the related genes through GeneMANIA. With the online tool KOBAS, KEGG pathway enrichment analysis on the downstream genes and their related genes was implemented, followed by the determination of related downstream pathways by looking up relevant literature.

### 2.3. Isolation and Identification of NPCs

Human normal NPCs were isolated by 0.2% collagenase digestion and subcultured utilizing monolayer adherence. Five cases of intraoperative IVDs of patients with fresh cervical vertebral fractures (no more than 3 days) were collected, including 2 C4-5 discs and 3 C5-6 discs. The samples were washed with D-Hank's solution three times in 0.5 h to remove blood stains. The annulus fibrosus, cartilage plate, and junction tissue were cut off, and the remaining jelly-like nucleus pulposus tissues were cut into 1 × 3 mm pieces with scissors and then digested with 0.2% type II collagenase for 4 h at 37°C. Digested tissues were centrifuged at 1000 × g for 5 min, with supernatant discarded. Cells in the lower layer were resuspended in DMEM-F12 medium containing 20% fetal bovine serum (FBS; 30067334, Thermo Fisher Scientific (China) Co., Ltd., Shanghai, China), seeded in 6 cm culture dish, and incubated at 37°C with 5% CO_2_. The medium was refreshed every 48 h with DMEM-F12 medium replenishing 10% FBS. Cell morphology was identified under a high magnification inverted microscope (EVOS XL Core, Thermo Fisher Scientific). After reaching 90% confluence in about 15 days, the cells were passaged at a ratio of 1 : 4 after trypsinization. Cells in passage 3 were used in this study.

Immunofluorescence was used to detect the expression of type II collagen (Col II), KRT-19, HIF-1*α*, SOX-9, and aggrecan (ACAN) in NPCs. In brief, cell slides were fixed in 4% paraformaldehyde at ambient temperature for 15 min and permeabilized with 0.5% Triton X-100 for 15 min. Next, the slide was blocked with 5% goat serum blocking solution for 30 min and probed with primary antibodies to HIF-*α* (ab51608, 1 : 500, Abcam, Cambridge, UK), KRT-19 (ab7754, 1 : 300, Abcam), SOX-9 (ab185966, 1 : 200, Abcam), Col II (ab34712, 1 : 200, Abcam), and ACAN (MA3-16888, 1 : 500, Invitrogen) at 4°C overnight. The next day, the cells were reprobed with fluorescently labeled secondary antibody IgG (mouse, ab150113, 1 : 200) or IgG (rabbit, ab150077, 1 : 200) at ambient temperature for 1 h and incubated with 4′,6-diamino-2-phenylindole (DAPI) (Sigma-Aldrich, St. Louis, MO, 1 : 2000) in the dark for 5 min, followed by observation under a microscope.

### 2.4. Transfection of NPCs

Logarithmically growing NPCs at passage 2 were trypsinized, seeded in 6-well plates (6 × 10^5^ cells/well), cultured at 37°C with 5% CO_2_, and transfected employing the Lipofectamine 3000 reagent (Gibco, Waltham, MA) with miR-31 mimic (B01001), mimic-NC (B04001), miR-31 inhibitor (B03001), and inhibitor-NC (B04003) (GenePharma, Shanghai, China). The NFAT5 coding sequence was amplified by EcoRI and XhoI (Thermo Fisher Scientific) double digestion and cloned into pcDNA3.1 (+) vector (Invitrogen, Carlsbad, CA). The cells were continuously cultured for 48 h and then used for subsequent experimentations. For simulating IDD *in vitro*, we used TNF-*α* to induce NPC apoptosis [26]. Cells were incubated for 48 h and then treated with TNF-*α* (5 ng/mL) for 12 h.

### 2.5. Isolation and Identification of BMSCs

Bone marrow specimens were provided by 3 hospitalized patients (2 males and 1 female, aged 26-52 years) with femur necrosis following MRI-based diagnosis at Tongji Hospital, Tongji Medical College, Huazhong University of Science and Technology. Patients with no loss of femoral head height were included while those with trauma, cardiovascular diseases, or tumor infiltration were excluded from this study. Human BMSCs were isolated from the bone marrow specimens of 3 donors as previously described [27] and cultured in DMEM-F12 medium (Hyclone, Logan, UT) with 10% fetal bovine serum (FBS, 10099141, Gibco) and 0.2% penicillin-streptomycin solution (Hyclone). hBMSCs were passaged every 3 days, and the cells at passage 3 were used for subsequent experimentations.

Next, the isolated hBMSCs were cultured in the OriCell™ MSC osteogenic, adipogenic, or chondrogenic differentiation medium (Cyagen, Guangzhou, China) for identification utilizing alizarin red staining, oil red O staining, and Alcian blue staining.

hBMSCs were trypsinized, centrifuged, and incubated with mouse monoclonal antibodies against CD105 (1 : 100, ab11414, Abcam), CD73 (1 : 50, ab239246, Abcam), CD90 (1 : 1000, ab23894, Abcam), CD45 (1 : 50, ab27287, Abcam), CD34 (1 : 50, ab131589, Abcam), CD14 (1 : 200, ab28061, Abcam), CD19 (1 : 50, ab134114, Abcam), murine monoclonal antibody HLA-DR (1 : 50, ab1182, Abcam), and FITC-conjugated goat anti-mouse IgG isotopic antibody (1 : 1000, BD Biosciences, San Jose, CA; serving as a negative control (NC)). Samples were quantified utilizing the FACSVerse system (BD Bioscience), and the results were analyzed by FlowJo software (Tree Star, Ashland, OR) to quantify the expression of surface antigens and nonsurface antigens of hBMSCs.

### 2.6. Isolation and Identification of BMSC-EVs

FBS was ultracentrifuged at 100,000 × g for 16 h at 4°C to remove EVs from the FBS. The obtained EV-depleted FBS was applied for this assay. After cell incubation for 72 h, the medium was collected and the EVs were separated by centrifugation (at 300 × g for 10 min, 2000 × g for 15 min, and 12000 × g for 30 min). Then, cells were passed through a 0.22 *μ*M filter. The supernatant was further ultracentrifuged at 1,000,000 × g for 2 h at 4°C, washed in PBS, and ultracentrifuged under the same conditions. Finally, EVs were resuspended in approximately 100 *μ*L PBS. EVs were isolated from the hBMSCs from three patients, mixed evenly, and stored at -80°C for standby or immediate use.

Particle size analysis was performed by NanoSight instrument (Salisbury, UK). The motion trajectory of each EV was analyzed and automatically converted into the diameter and concentration of EVs according to the principle of Brownian motion, which can be converted into the original concentration according to the dilution ratio [28]. A Hitachi H7650 transmission electron microscope (Tokyo, Japan) was adopted to study the characterization of EVs [29].

### 2.7. Treatment of EVs with Proteinase K and RNase A

The isolated BMSC-EVs were incubated with proteinase K (0.05 *μ*g/*μ*L; Sigma Aldrich) for 10 min at 37°C and then with 5 mM phenylmethylsulfonyl fluoride (PMSF; Sigma Aldrich) for 10 min at ambient temperature to limit proteinase K activity, followed by complete inactivation of proteinase K by heating at 90°C for 5 min. Following this, samples were incubated with RNase A at a final concentration of 0.5 *μ*g/*μ*L (Thermo Fisher Scientific) for 20 min at 37°C to digest the exposed RNA. In the control group, proteinase K, PMSF, or RNase A was substituted by the same amount of PBS. RNA was finally extracted and used for subsequent analysis.

### 2.8. EV Uptake by NPCs

Purified EVs were labeled with a PKH67 green fluorescence kit (Sigma-Aldrich). EVs were resuspended in 1 mL of diluent C solution. PKH-67 ethanol dye solution (4 *μ*L) was added to 1 mL of diluent C to prepare a 4 × 10^−6^ M dye solution. EV suspension was mixed with the dye solution for 5 min. BSA (1%, 2 mL) was added for 1 min to stop staining. The labeled EVs were ultracentrifuged at 100,000 × g for 70 min, washed with PBS, ultracentrifuged again, and resuspended in 50 *μ*L PBS. EVs were cocultured with NPCs for 12 h at 37°C, and the NPCs were fixed with 4% paraformaldehyde after which nuclei were stained with DAPI. The uptake of labeled EVs by NPCs was measured with a fluorescence microscope (Zeiss, Oberkochen, Germany).

### 2.9. Lentivirus Transduction of hBMSCs

BMSCs were seeded in 6-well plates at 6.0 × 10^5^ cells/well and transduced with lentivirus carrying miR-31-knockdown (anti-miR-31; GL–02; GeneCopoeia, Rockville, MD) and negative control (anti-NC; GL–02; GeneCopoeia, Rockville, MD) using a Lentiviral Transduction Kit (MOI = 50), followed by culture at 37°C in 5% CO_2_ for 72 h.

The uptake of EVs from BMSCs carrying Cy3-miR-31 by recipient cells (NPCs) was conducted. Briefly, hBMSCs were transfected with Cy3-miR-31 mimic (GenePharma) employing the Lipofectamine 3000 reagent (Invitrogen, L3000001). Six h later, the serum-free medium was changed with the 10% EV- and serum-free medium, and cells were cultured for 48 h. Cell supernatant was collected, resuspended in PBS, and added to NPCs. With the same method, cells were fixed with 4% paraformaldehyde, and the cytoskeleton was labeled with Phalloidin-iFLuor 488 (1 : 1000, #ab176753, Abcam, green fluorescence) at ambient temperature for 30 min. Nuclei were stained with DAPI (D9542, Sigma-Aldrich). NPCs (green) under a microscope or a confocal microscope (LSM 710, Zeiss) were observed to internalize the EVs (red) from BMSCs carrying Cy3-miR-31 [30, 31].

### 2.10. Coculture of NPCs with hBMSCs or with EVs

The transwell system (6-well plate with the diameter of a single well of 12 mm) was used to coculture NPCs and hBMSCs indirectly. hBMSCs (2 × 10^5^ cells/well) were settled in the upper chamber while NPCs (2 × 10^5^ cells/well) were in the lower chamber for 14 days of coculture (about 60% confluence). During the coculture of NPCs with BMSC-EVs, BMSC-EVs (final concentration of 50 *μ*g/mL) were directly added to the NPC medium, and PBS was used as a control for BMSC-EVs. NPCs were mainly grouped into PBS, TNF-*α* (NPCs were exposed to 5 ng/mL TNF-*α*), hBMSCs (NPCs were cocultured with hBMSCs), GW4869 (NPCs were treated with 5 *μ*M EV inhibitor GW4869), hBMSCs-EVs (NPCs were cocultured with hBMSC-EVs), hBMSCs-EVs NC inhibitor (NPCs were cocultured with EVs from hBMSCs transduced with lentivirus carrying NC inhibitor), and hBMSCs-EVs anti-miR-31 (NPCs were cocultured with EVs from hBMSCs transduced with lentivirus carrying miR-31-knockdown).

### 2.11. Dual Luciferase Reporter Assay

HEK293T cells (ATCC) were cultured in 48-well plates for 24 h. pRL-TK luciferase reporter plasmid (Promega, Madison, WI) was used to construct NFAT5 wild type (WT) 3′UTR or mutant (Mut) plasmids, which were then cotransfected with 50 nmol/L miR-31 mimic or mimic-NC into HEK293T cells for 48 h. The Dual Luciferase Reporter Assay System (Promega) was employed to quantify the relative luciferase activity, with Renilla luciferase used as an internal reference.

### 2.12. TOPFlash

Cells were seeded into 96-well plates, cultured for 24 h, and transfected with plasmids, TOPFlash or FOPFlash, and internal reference pRL-TK plasmids (Promega) by referring to Lipofectamine 2000 reagent instructions (11668019, Thermo Fisher Scientific). The plasmids were then mixed with 100 *μ*L L-DMEM and left to stand at ambient temperature for 5 min. Next, 0.5 *μ*L Lipofectamine 2000 was mixed with 100 *μ*L L-DMEM and left to stand at ambient temperature for 5 min. Next, the culture medium was washed off, and cells were washed with L-DMEM and transfected with the mixed transfection medium for 6 h, followed by culture in the renewed complete culture medium. After 24-48 h, the culture medium was discarded, and the Dual Luciferase Reporter Assay System (E1910, Promega) was adopted to quantify the activities of Renilla and Firefly luciferase in each well. The ratio of the two reflects the activation level of transcription factors in the intracellular Wnt/*β*-catenin pathway [32].

### 2.13. CCK-8 Assay

The CCK-8 kit (C0037, Beyotime, Shanghai, China) was adopted to test the effects of hBMSC-EVs on the proliferation of NPCs. Cells were seeded at 2 × 10^3^ cells/well in 96-well plates. After 24 h, 10 *μ*L of the CCK-8 reagent was added to 100 *μ*L of complete culture medium at different time points (0, 24, 36, 48, and 72 h) and continued to incubate for 4 h. The OD value of each well was quantified at 450 nm utilizing a Multiskan FC microplate reader (51119100, Thermo Fisher Scientific).

### 2.14. Flow Cytometry

Apoptosis was checked with the help of the Annexin V-FITC/PI staining kit (BD Biosciences). NPCs were seeded in a 6-well plate and treated with PBS or hBMSC-EVs for 24 h upon reaching 70% confluence. Next, the cells were digested with trypsin without EDTA and stained with Annexin V-FITC/PI. After 15 min, the cells were detected utilizing a flow cytometer (BD FACSVerse™; BD Bioscience). Fluorescence was initiated by excitation at 488 nm (FITC) and 535 nm (PI) and was evaluated utilizing emission filters at 525 nm (FITC) and 615 nm (PI).

### 2.15. Establishment of IDD Models in Mice

C57BL/6J mice (*n* = 60, 2-3 months old, 18-20 g; Beijing Vital River Laboratory Animal Technology Co., Ltd., Beijing, China) were equilibrated for 1 week, followed by tail needle puncture to mimic IDD in vivo [7, 33]. Needle puncture was performed under general anesthesia (2% isoflurane oxygen) and sterile conditions. Caudal IVDs (cc4-5 and cc6-7) were exposed through palpation and a 24 mm dorsal lateral incision. After determining the location of IVDs microscopically (M60, Leica Microsystems, IL), a 26 G needle was used to puncture 50% of the width of the back. The wound was closed by sutures (Prolene 8-0 sutures). Mice were intraperitoneally injected with penicillin sodium 200,000 U/kg (North China Pharmaceutical, Shijiazhuang, Hebei, China) once a day for 3 days to prevent infection. Mice were housed at 23-25°C separately and fed with standard chow. The success rate was 96% (48/50).

One week after the operation, mice were randomized into (10 mice in each group) control (mouse skin was cut and then immediately sutured), IDD (mice following IDD model construction), IDD+saline (IDD mice injected with equal volume of sterile saline), IDD+hBMSCs-EVs (IDD mice injected with hBMSC-EVs), IDD+hBMSCs-EVs anti-NC (IDD mice injected with hBMSCs-EVs anti-NC), and IDD+hBMSCs-EVs anti-miR-31 (IDD mice injected with hBMSCs-EVs anti-miR-31). IDD mice were anesthetized, and a small incision was prepared to expose the previously punctured IVDs. A total of 50 *μ*L of sterile saline containing different purified EVs (approximately 1.5 × 10^6^) was injected slowly into the punctured disc site using a 0.38 mm × 8 mm syringe. The injection was repeated 4 weeks later. After 9 weeks, MRI was used to detect the puncture IVD of mice after the operation, and then, all mice were euthanized with CO_2_. IVD tissues were harvested and stored for subsequent experiments. To track the distribution of EVs in the mouse, EVs were prelabeled with PKH26.

### 2.16. Pathological Analysis

IVD tissues were fixed in 4% paraformaldehyde for 48 h, decalcified in 25% formic acid and 10% sodium citrate for 2 d, embedded in paraffin, and cut into 5 *μ*m sections. Sections were subjected to hematoxylin-eosin (HE) or TUNEL staining. Histological image analysis was completed under an optical microscope (DMM-300D, Caikon, Shanghai, China). The grading score for HE staining was performed by referring to the criteria established previously [34]. Apoptotic activity was determined by an in situ luciferin cell death detection kit (Roche, Mannheim, Germany). TUNEL-positive cells were analyzed by Image-Pro Plus software (Media Cybernetics, Silver Spring, MA). Data collection and analysis were completed by two independent investigators blinded to experimental groups.

### 2.17. ELISA

Serum of mice was collected, and the levels of TNF-*α* and IL-1*β* were detected by the ELISA Kit (DTA00D, DLB50, R&D, Minneapolis, MN).

### 2.18. RT-qPCR

Total RNA from cells or tissues was extracted with the TRIzol reagent (Invitrogen) and then reversely transcribed into cDNA using the PrimeScript RT kit (Takara, Kusatsu, Japan). For miRNA detection, the RNA was reverse transcribed into cDNA using the miRcute Plus miRNA first-strand cDNA synthesis kit (TianGen, Beijing, China). miRNA in the medium (350 *μ*L) and EVs (100 *μ*g) was extracted with the help of the mirVana PARIS Kit (Ambion, Austin, TX). An exogenous reference cel-miR-39 (1 pmol/sample; TianGen) was added. RT-qPCR for mRNA was performed using the SYBR Premix Ex Taq Reagent Kit (Takara) and ABI StepOne real-time PCR system (Applied Biosystems, Foster City, CA). RT-qPCR for miRNA was processed utilizing the miRcute Plus miRNA qPCR Detection Kit (TianGen). *β*-Actin was used as a normalizer for mRNA while U6 for miRNA. In addition, miRNA expression in culture media and EVs was normalized to the exogenous reference cel-miR-39. The relative expression of target genes was calculated by the 2^*ΔΔ*CT^ method. Primer sequences are described in Supplementary Table 1.

### 2.19. Western Blot

EV markers were determined by resuspending hBMSC-EVs in precooled protease inhibitor (Roche)-containing lysis buffer. The lysate was dissolved in 3× Laemmli's sample buffer, heated for 5 min, separated with 12% SDS-PAGE, and transferred to a nitrocellulose membrane. The membrane was blocked with 5% nonfat milk in pH 7.4 phosphate buffer (137 mmol/L NaCl, 2.7 mmol/L KCl, 10 mmol/L Na_2_HPO_4_, and 2 mmol/L KH_2_PO_4_) and 0.05% Tween. Next, the membrane was incubated with primary rabbit antibodies against tumor susceptibility gene 101 (TSG101) (1 : 1000, ab30871, Abcam), CD63 (1 : 1000, ab216130, Abcam), ALIX (1 : 1000, ab88743, Abcam), and GRP94 (1 : 1000, ab13509, Abcam) and then with horseradish peroxidase-conjugated secondary antibody (1 : 5000, ab6721, Abcam). The membrane was visualized employing the enhanced chemiluminescence reagent (170-8280, Bio-Rad Laboratories, Hercules, CA). Ponceau red was used as an internal reference. Data analysis was completed utilizing ImageJ software (National Institutes of Health, Bethesda, MD).

Protein expression was also determined in cell and tissue lysate. Protein concentration was determined employing a Bradford assay (Bio-Rad). Protein was then separated with 10% SDS-PAGE and transferred to a nitrocellulose membrane. Next, the membrane was incubated with primary antibodies against NFAT5 (ab3446, Abcam), *β*-catenin (ab6302, Abcam), cleaved caspase-3 (ab2302, Abcam), Bcl-2 (ab59348, Abcam), Bax (ab32503, Abcam), ACAN (ab3778, Abcam), Col II (ab34712, Abcam), SOX-9 (ab26414, Abcam), MMP-3 (ab52915, Abcam), TIMP-1 (ab61224, Abcam), and Nanog (1 : 1000, ab109250, Abcam). GAPDH (1 : 5000, 5174, Cell Signaling Technology) was used as an internal reference.

### 2.20. Statistical Analysis

Statistical analysis was completed with the help of SPSS 21.0. Data were expressed as the mean ± standard deviation. Data obeying normal distribution and homogeneity of variance between two groups were compared by the unpaired *t*-test. Data comparison among multiple groups was started by one-way ANOVA and Tukey's post hoc test. Data comparison between groups at different time points was processed by repeated measures ANOVA and Bonferroni's post hoc test. In the samples with skewed distribution or defect variances, the rank-sum test was used. Differences were deemed significant when *p* < 0.05.

## 3. Results

### 3.1. hBMSC-EVs Deliver miR-31

Flow cytometric data revealed that the expression of BMSC surface antigens CD73, CD90, and CD105 was higher than 95% of all cells, while that of non-BMSC surface antigens CD34, CD45, CD14, CD16, and HLA-DR was less than 2% (Supplementary Figure 1A). These results suggested that the isolated hBMSCs were of high purity. In addition, under an optical microscope, hBMSCs were spindle-shaped and grow in colonies. After differentiation induction under different conditions, red calcium nodules, red lipid-like cells, and blue collagen staining were observed following alizarin red staining, oil red O staining, and Alcian blue staining, respectively, suggesting that hBMSCs had osteogenic, adipogenic, and chondrogenic differentiation potentials (Supplementary Figure 1B). Furthermore, the expression of BMSC stemness marker protein Nanog in hBMSCs at passage 3 was basically the same as that in the hBMSCs at passage 1 (Supplementary Figure 1C), which indicated that the BMSCs at passage 3 still retain the stemness of BMSCs when we used the BMSCs at passage 3 for experiments.

**Figure 1:**
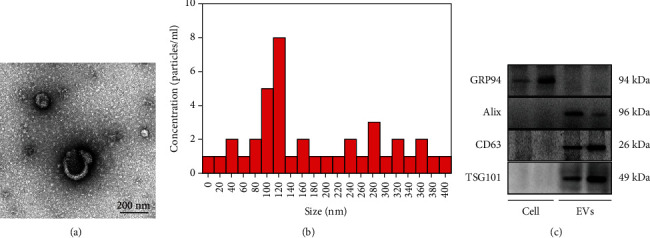
Characterization of hBMSC-EVs: (a) transmission electronic microscopic image of EVs, scale bar = 200 nm; (b) size of EVs determined by NanoSight; (c) protein expression of ALIX, CD63, TSG101, and GRP94 in the isolated hBMSC-EVs determined by Western blot.

Under an electron microscope, the EVs had saucer-like structures with clear membranes, about 200 nm (Figure 1(a)). EVs derived from hBMSCs had diameters ranging from 100 to 400 nm, with the highest concentration of 1.8 particles/mL (Figure 1(b)). Furthermore, the results of Western blot showed the presence of expression of EV marker proteins ALIX, CD63, and TSG101 in the isolated BMSC-EVs, but no endoplasmic reticulum-associated protein GRP94 (Figure 1(c)).

**Figure 2:**
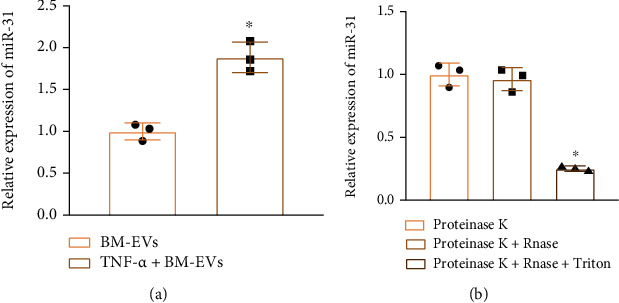
miR-31 expression in the isolated hBMSC-EVs: (a) miR-31 expression in the isolated hBMSC-EVs after TNF-*α* treatment determined by RT-qPCR; (b) miR-31 expression in the isolated hBMSC-EVs after RNAse or proteinase K treatment determined by RT-qPCR. ^∗^*p* < 0.05 vs. hBMSC-EVs or proteinase K+RNAse. Cell experiments were repeated three times.

After TNF-*α* exposure, miR-31 expression was increased in the supernatant of BMSC-EVs (Figure 2(a)). Moreover, the combined use of proteinase K and RNase did not reduce the level of miR-31 in EVs; only when the EV membrane was damaged by Triton and subjected to the action of RNase was the expression of miR-31 reduced (Figure 2(b)). These results indicated that BMSCs could secrete EVs encapsulating miR-31.

### 3.2. hBMSC-EVs Deliver miR-31 to NPCs

**Figure 3:**
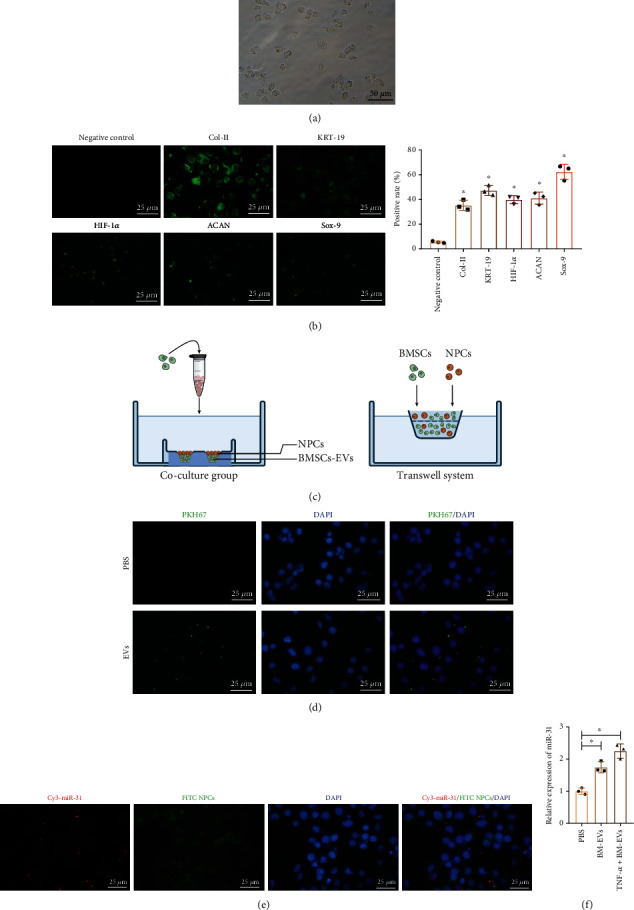
hBMSC-EVs deliver miR-31 to NPCs: (a) microscopic images of NPCs. Scale bar = 50 *μ*m. (b) Immunofluorescence detection of Col II, KRT-19, HIF-1*α*, SOX-9, and ACAN proteins in the NPCs. Scale bar = 25 *μ*m. (c) Diagram showing coculture of hBMSCs or hBMSC-EVs with NPCs. (d) Confocal microscopy was used to observe the uptake of PKH-67 labeled EVs by NPCs after coculture for 24 h. Scale bar = 25 *μ*m. (e) Representative fluorescence micrographs in NPCs cocultured with Cy3-miR-31-BMSCs (red). Scale bar = 25 *μ*m. (f) miR-31 expression in NPCs cocultured with BMSC-EVs or TNF-*α*+BMSC-EVs determined by RT-qPCR. ^∗^*p* < 0.05. Cell experiments were repeated three times.

In order to explore the potential function of miR-31 delivered by hBMSC-EVs in NPCs, we firstly isolated NPCs and observed the morphology of NPCs using a microscope. The results showed that the shape of NPCs was fusiform or multiangle, and the process of cytoplasm was long (Figure 3(a)). The purified NPCs were identified by immunofluorescence. The results showed that Col II, KRT-19, HIF-1*α*, SOX-9, and ACAN expressions were positive in the NPCs at passage 2 (Figure 3(b)), demonstrating the successful isolation of NPCs. PKH67-labeled hBMSC-EVs (green) were cocultured with hBMSCs transfected with the Cy3-labeled miR-31 (red; Cy3-miR-31-BMSCs) (Figure 3(c)). NPCs showed green fluorescence following coculture with EVs (Figure 3(d)) and red fluorescence after coculture with Cy3-miR-31-BMSCs (Figure 3(e)), suggesting that hBMSC-EVs could carry miR-31 and deliver it into NPCs. Moreover, increased miR-31 expression was found in NPCs cocultured with hBMSC-EVs, and the addition of TNF-*α* further increased miR-31 expression in NPCs cocultured with hBMSC-EVs (Figure 3(f)). Thus, these data revealed that hBMSC-EVs could transfer miR-31 to NPCs.

### 3.3. hBMSC-EVs Promote NPC Proliferation but Inhibit NPC Apoptosis and ECM Degradation

**Figure 4:**
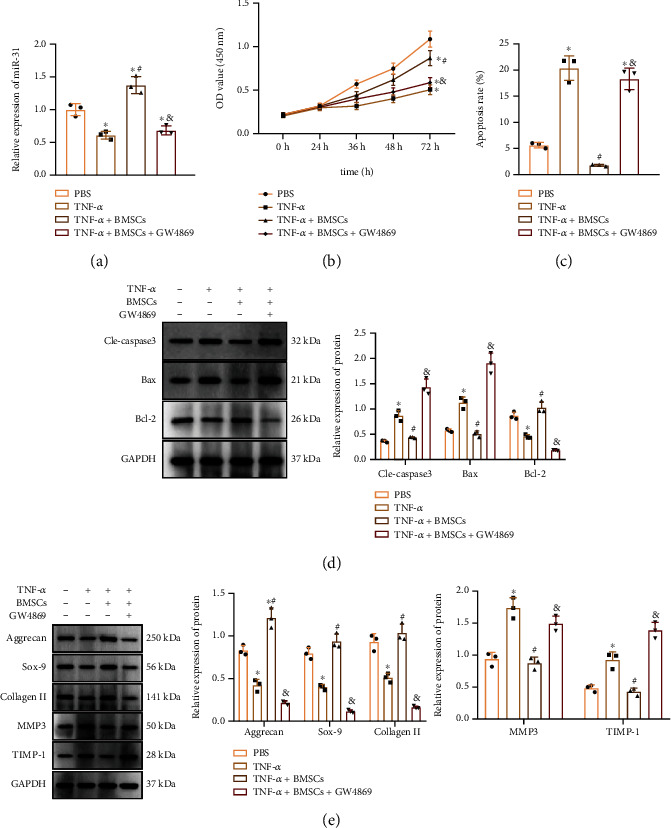
hBMSC-EVs inhibit NPC apoptosis and ECM degradation and promote cell proliferation in NPCs. (a) miR-31 expression in TNF-*α*-treated NPCs cocultured with BMSCs or further treated with GW4869 determined by qRT-PCR. (b) Proliferation of TNF-*α*-treated NPCs cocultured with BMSCs or further treated with GW4869 determined by CCK-8 assay. (c) Apoptosis of TNF-*α*-treated NPCs cocultured with BMSCs or further treated with GW4869 determined by flow cytometry. (d) Protein expression of apoptosis-related genes cleaved caspase-3, Bax, and Bcl-2 in TNF-*α*-treated NPCs cocultured with BMSCs or further treated with GW4869 determined by Western blot. (e) Protein expression of ECM-related genes in TNF-*α*-treated NPCs cocultured with BMSCs or further treated with GW4869 determined by Western blot. ^∗^*p* < 0.05 vs. PBS; ^#^*p* < 0.05 vs. TNF-*α*; ^&^*p* < 0.05 vs. TNF-*α*+hBMSCs. Cell experiments were repeated three times.

The effect of BMSC-EVs delivering miR-31 on NPCs was subsequently evaluated. TNF-*α* treatment reduced miR-31 expression in NPCs (Figure 4(a)), cell proliferation (Figure 4(b)), and increased apoptosis (Figure 4(c)). TNF-*α* treatment also increased cleaved caspase-3 and Bax expression while decreasing Bcl-2 expression in NPCs (Figure 4(d)). In addition, TNF-*α* treatment in NPCs reduced the expression of ECM synthesis-related genes (ACAN, Col II, and SOX-9), while increasing that of ECM degradation-related genes MMP-3 and TIMP-1 (Figure 4(e)). However, the addition of BMSCs reversed the above effects of TNF-*α*, but further treatment with GW4869 caused similar results to those of TNF-*α* (Figures 4(a)–4(e)). These results showed that hBMSC-EVs may promote NPC proliferation and inhibit cell apoptosis and ECM degradation.

### 3.4. miR-31 Delivered by hBMSC-EVs Promotes NPC Proliferation while Reducing Cell Apoptosis and ECM Degradation

**Figure 5:**
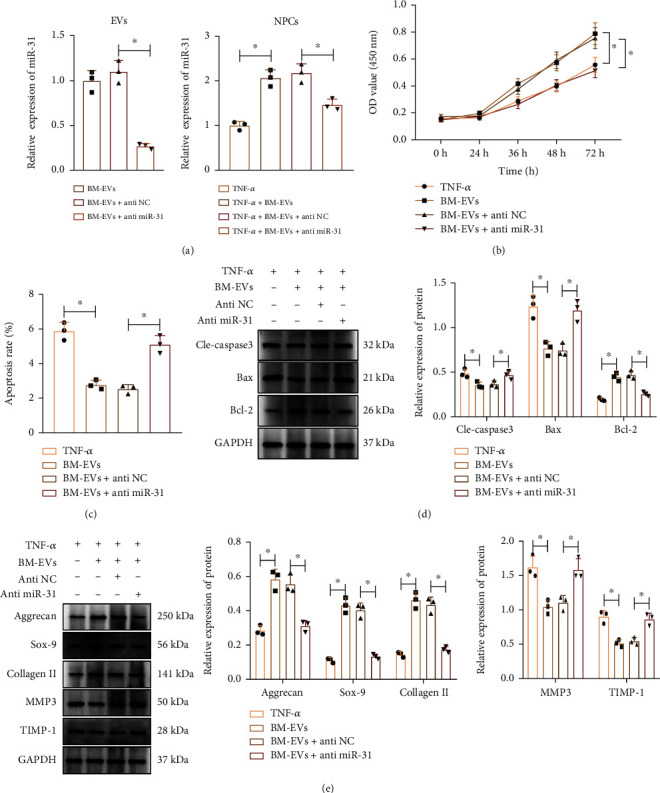
miR-31 in hBMSC-EVs augments NPC proliferation and inhibits cell apoptosis and ECM degradation in NPCs. (a) miR-31 expression in EVs from hBMSCs transduced with lentivirus carrying anti-miR-31 (left) and in TNF-*α*-treated NPCs cocultured with BMSCs-EVs+anti-miR-31 (right) determined by RT-qPCR. (b) Proliferation of TNF-*α*-treated NPCs cocultured with BMSCs-EVs+anti-miR-31 determined by CCK-8 assay. (c) Apoptosis of TNF-*α*-treated NPCs cocultured with BMSCs-EVs+anti-miR-31 determined by flow cytometry. (d) Protein expression of apoptosis-related genes cleaved caspase-3, Bax, and Bcl-2 in TNF-*α*-treated NPCs cocultured with BMSCs-EVs+anti-miR-31 determined by Western blot. (e) Protein expression of ECM-related genes in TNF-*α*-treated NPCs cocultured with BMSCs-EVs+anti-miR-31 determined by Western blot. ^∗^*p* < 0.05. Cell experiments were repeated three times.

Next, we sought to determine whether BMSC-EVs stimulated NPC proliferation and repressed cell apoptosis by delivering miR-31 to NPCs. Coculture with hBMSC-EVs in TNF-*α*-treated NPCs increased miR-31 expression (Figure 5(a)) and cell proliferation (Figure 5(b)), but repressed apoptosis (Figure 5(c)), decreased cleaved caspase-3 and Bax expression, and increased Bcl-2 expression (Figure 5(d)) when compared to TNF-*α* treatment alone. In addition, hBMSC-EVs also increased the expression of ACAN, Col II, and SOX-9, while decreasing that of MMP-3 and TIMP-1 (Figure 5(e)). In contrast, BMSCs-EVs+anti-miR-31 in TNF-*α*-treated NPCs reversed the effects of hBMSC-EVs (Figures 5(a)–5(e)). Altogether, the above results indicated that hBMSCs transferred miR-31 to NPCs where miR-31 induced NPC proliferation and inhibited cell apoptosis and ECM degradation.

### 3.5. miR-31 Targets NFAT5 and Activates the Wnt/*β*-Catenin Pathway

**Figure 6:**
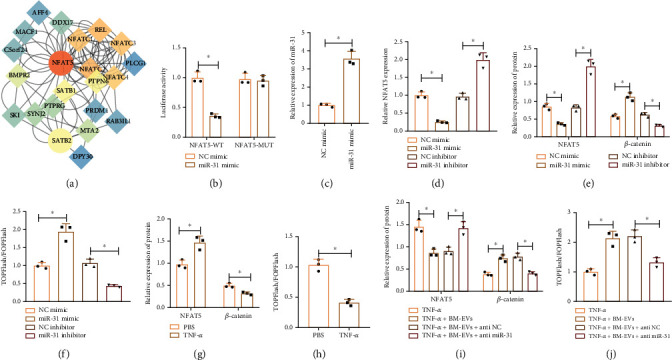
miR-31 targets NFAT5 and activates the Wnt/*β*-catenin pathway in NPCs: (a) a PPI network of the genes related to the key downstream genes constructed through GeneMANIA; (b) binding of miR-31 to NFAT5 in 293T cells determined by dual luciferase reporter assay; (c) expression of miR-31 in NPCs after transfection with miR-31 mimic or miR-31 inhibitor determined by RT-qPCR; (d) expression of NFAT5 in NPCs after transfection with miR-31 mimic or miR-31 inhibitor determined by RT-qPCR; (e) expression of NFAT5 and *β*-catenin in NPCs after transfection with miR-31 mimic or miR-31 inhibitor determined by Western blot; (f) the transcription activity of TCF/LEF in NPCs after transfection with miR-31 mimic or miR-31 inhibitor determined by TOPFlash; (g) protein expression of NFAT5 and *β*-catenin in TNF-*α*-treated NPCs determined by Western blot; (h) the transcription activity of TCF/LEF in TNF-*α*-treated NPCs determined by TOPFlash; (i) expression of NFAT5 and *β*-catenin in TNF-*α*-treated NPCs cocultured with BMSCs-EVs or BMSCs-EVs+anti-miR-31 determined by Western blot; (j) the transcription activity of TCF/LEF in TNF-*α*-treated NPCs cocultured with BMSCs-EVs or BMSCs-EVs+anti-miR-31 determined by TOPFlash. ^∗^*p* < 0.05. Cell experiments were repeated three times.

The Venn diagram of the downstream genes of miR-31 predicted by the RAID, TargetScan, mirDIP, and miRWalk databases suggested that SATB2 and NFAT5 were at the intersection (Supplementary Figure 2A). GeneMANIA was used to predict the genes related to the key downstream genes, followed by the construction of a PPI network. Then, Cytoscape was adopted to calculate the core degree with the results presenting that the core degree of NFAT5 was 32 (Figure 6(a)). Meanwhile, evidence has indicated that NFAT5 can promote the occurrence of IDD [35]. Thus, NFAT5 was selected for subsequent experiments.

The TargetScan database predicted the binding sites of miR-31 in the NFAT5 3′UTR in humans, mice, and rats (Supplementary Figure 2B). The dual luciferase reporter assay further verified that the luciferase activity of NFAT5 WT 3′UTR was decreased in response to transfection with miR-31 mimic, but that of NFAT5 MUT 3′UTR was unaffected (Figure 6(b)). Moreover, miR-31 mimic elevated miR-31 expression but reduced NFAT5 expression, while miR-31 inhibitor exerted opposite results (Figures 6(c) and 6(d)). These results suggested that miR-31 targeted NFAT5 3′UTR and limited its expression.

GeneMANIA predicted 20 genes related to NFAT5 (Supplementary Figure 3A). Enrichment analysis of NFAT5 and its related genes using KOBAS showed that related genes were mainly enriched in the Wnt pathway (Supplementary Figure 3B). Previous studies have shown that NFAT5 inhibits activation of the Wnt pathway [18], and the Wnt pathway can inhibit the occurrence of IDD [21]. Therefore, we speculated that miR-31 targeted NFAT5 and mediated the Wnt pathway to regulate IDD. To test this conjecture, Western blot was first conducted to detect the expression of the Wnt/*β*-catenin pathway-related proteins in the presence of miR-31 mimic or inhibitor. The results described that miR-31 mimic decreased NFAT5 expression and increased *β*-catenin expression (Figure 6(e)). In contrast, miR-31 inhibitor led to opposite results (Figure 6(e)). In addition, TOPFlash results showed that the transcription activity of TCF/LEF was increased by miR-31 mimic, but decreased by miR-31 inhibitor (Figure 6(f)).

Furthermore, TNF-*α* treatment increased NFAT5 protein expression, decreased *β*-catenin expression, and reduced transcription activity of TCF/LEF in NPCs (Figures 6(g) and 6(h)), while BMSCs-EVs had opposite effects (Figures 6(i) and 6(j)). Furthermore, compared with BMSCs-EVs+anti-NC, BMSCs-EVs+anti-miR-31 treatment increased NFAT5 protein expression, decreased *β*-catenin expression, and reduced transcription activity of TCF/LEF in NPCs (Figures 6(i) and 6(j)). The aforementioned results indicated that miR-31 targeted NFAT5, upregulated the expression of *β*-catenin protein, and activated the Wnt/*β*-catenin pathway.

### 3.6. miR-31 in hBMSC-EVs Targets NFAT5 to Repress NPC Apoptosis and ECM Degradation while Stimulating Cell Proliferation

**Figure 7:**
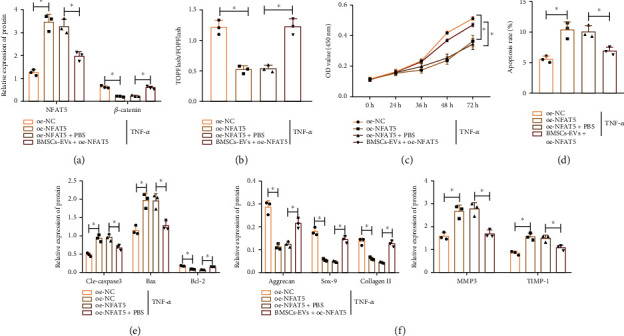
miR-31 in hBMSC-EVs targets NFAT5 to inhibit NPC apoptosis and ECM degradation in NPCs: (a) protein expression of NFAT5 and *β*-catenin in TNF-*α*-exposed NPCs treated with oe-NFAT5 or combined with BMSCs-EVs determined by Western blot; (b) the transcription activity of TCF/LEF in TNF-*α*-exposed NPCs treated with oe-NFAT5 or combined with BMSCs-EVs determined by TOPFlash; (c) proliferation of TNF-*α*-exposed NPCs treated with oe-NFAT5 or combined with BMSCs-EVs determined by CCK-8 assay; (d) apoptosis of TNF-*α*-exposed NPCs treated with oe-NFAT5 or combined with BMSCs-EVs determined by flow cytometry; (e) protein expression of apoptosis-related genes cleaved caspase-3, Bax, and Bcl-2 in TNF-*α*-exposed NPCs treated with oe-NFAT5 or combined with BMSCs-EVs determined by Western blot; (f) protein expression of ECM-related genes in TNF-*α*-exposed NPCs treated with oe-NFAT5 or combined with BMSCs-EVs determined by Western blot. ^∗^*p* < 0.05. Cell experiments were repeated three times.

The aforementioned results allowed us to speculate that the effect of miR-31 in hBMSC-EVs on the NPC biological function was associated with NFAT5. Treatment with oe-NFAT5 in TNF-*α*-treated NPCs increased NFAT5 protein expression, decreased *β*-catenin protein expression (Figure 7(a)), reduced the transcription activity of TCF/LEF (Figure 7(b)), decreased cell proliferation (Figure 7(c)), and increased cell apoptosis (Figure 7(d)). In addition, NFAT5 overexpression also increased cleaved caspase-3 and Bax protein expression but diminished Bcl-2 protein expression (Figure 7(e)). NFAT5 overexpression reduced the protein expression of ACAN, Col II, and SOX-9 while increasing that of MMP-3 and TIMP-1 (Figure 7(f)). Conversely, the addition of hBMSC-EVs reversed the effects of NFAT5 overexpression (Figures 7(a)–7(f)). These lines of evidence demonstrated that miR-31 in hBMSC-EVs inhibited the expression of NFAT5 in NPCs, thereby accelerating NPC proliferation and inhibiting NPC apoptosis and ECM degradation.

### 3.7. miR-31 in hBMSC-EVs Alleviates IDD in Mice

**Figure 8:**
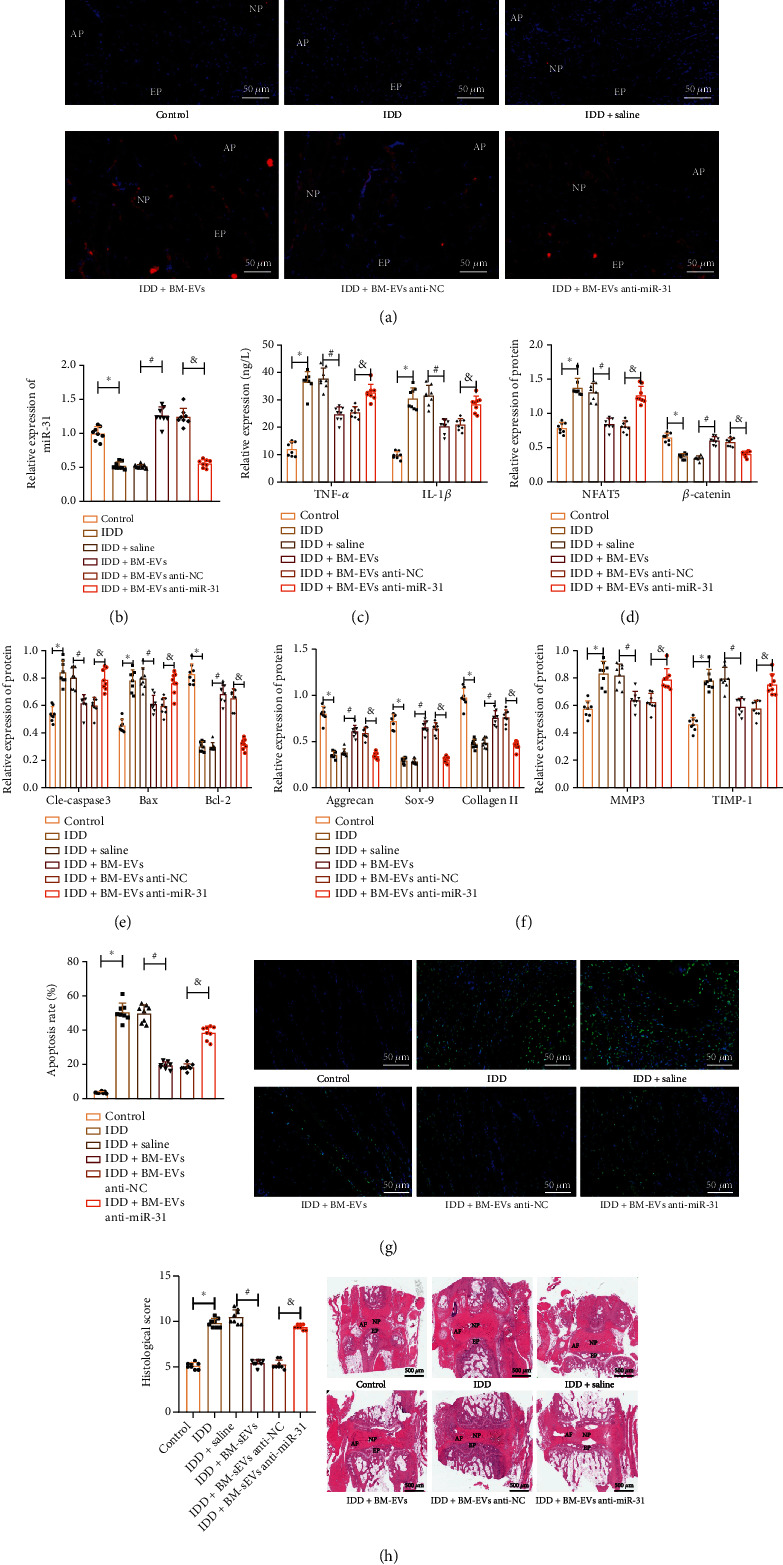
miR-31 in hBMSC-EVs relieves IDD in mice: (a) representative fluorescence micrographs showing PKH26-labeled EVs in IVD tissues of IDD mice treated with BMSCs-EVs or combined with anti-miR-31. Scale bar = 50 *μ*m. (b) miR-31 expression in IVD tissues; (c) serum levels of inflammatory factors in IDD mice treated with BMSCs-EVs or combined with anti-miR-31 determined by ELISA; (d) protein expression of NFAT5 and *β*-catenin in IVD tissues of IDD mice treated with BMSCs-EVs or combined with anti-miR-31 determined by Western blot; (e) protein expression of cleaved caspase-3, Bax, and Bcl-2 in IVD tissues of IDD mice treated with BMSCs-EVs or combined with anti-miR-31 determined by Western blot; (f) protein expression of ECM-related genes in IVD tissues of IDD mice treated with BMSCs-EVs or combined with anti-miR-31 determined by Western blot; (g) cell apoptosis determined by TUNEL assay in IVD tissues of IDD mice treated with BMSCs-EVs or combined with anti-miR-31 9 weeks after puncture. Blue fluorescence (DAPI) indicates the total number of cells; green fluorescence (FITC) indicates TUNEL-positive cells. Scale bar = 50 *μ*m. (h) HE staining of IVD tissues of IDD mice treated with BMSCs-EVs or combined with anti-miR-31. Scale bar = 500 *μ*m. ^∗^*p* < 0.05.*n* = 8.

We finally aimed to characterize the effect of miR-31 in hBMSC-EVs on IDD *in vivo*. PKH26-labeled hBMSC-EVs were distributed in NP tissues (Figure 8(a)). miR-31 expression was reduced in the IVD tissues of IDD mice (Figure 8(b)). Serum levels of TNF-*α* and IL-1*β* were found to be increased in IDD mice (Figure 8(c)). In addition, the protein expression of NFAT5, cleaved caspase-3, Bax, MMP-3, and TIMP-1 was elevated while that of *β*-catenin, Bcl-2, ACAN, Col II, and SOX-9 was decreased in the IVD tissues of IDD mice (Figures 8(d)–8(f)).

Additionally, an increase was noted in the cell apoptosis (Figure 8(g)) and histological score (Figure 8(h)) in the IVD tissues of IDD mice. The addition of hBMSC-EVs reversed the above changes in IDD mice (Figures 8(a)–8(h)). However, anti-miR-31 abrogated the effect of hBMSCs-EVs (Figures 8(a)–8(h)). These results indicated that miR-31 in BMSC-EVs alleviated IDD in mice.

## 4. Discussion

There are a number of important findings in this study. First, we found that hBMSC-EVs carried and delivered miR-31 into NPCs. TNF-*α* treatment significantly decreased the expression of miR-31 in NPCs. Second, hBMSC-EVs decreased NPC apoptosis and ECM degradation. Third, we further demonstrated that miR-31 also decreased NPC apoptosis and ECM degradation, which was blocked by miR-31 inhibitor. Fourth, miR-31 bound to and inhibited NFAT5, leading to increased *β*-catenin and activation of the Wnt/*β*-catenin pathway. TNF-*α* treatment also had similar effects that were inhibited by hBMSC-EV treatment. Fifth, NFAT5 overexpression increased NPC apoptosis and ECM degradation, suggesting NFAT5 promoted the progression of IDD. These effects of NFAT5 were inhibited by hBMSC-EV treatment. Lastly, miR-31 expression was decreased in the IVD tissues of IDD mice, together with increased proinflammatory cytokines, ECM degradation, and NPC apoptosis. These effects can be alleviated by hBMSC-EV treatment. Meanwhile, the effect of hBMSC-EV treatment was blocked by miR-31 inhibitor. These results strongly suggested that miR-31 in hBMSC-EVs inhibited NFAT5, leading to activation of the Wnt/*β*-catenin pathway and alleviating IDD.

Accumulating evidence has documented the increased degradative enzymes and proinflammatory cytokines in IDD [6, 36, 37]. Notably, apoptosis of NPCs is a major cause of IDD [7]. One of the most important findings in this study was that miR-31 was delivered by hBMSC-EVs into NPCs. EVs are well known for cell-to-cell communications through proteins, lipids, or RNAs [9]. miR-31 has been confirmed to be encapsulated in synovial MSC-EVs and can be transferred into the target chondrocytes [10]. Besides, miR-31-5p is highly expressed in MSC-exosomes, which can deliver miR-31-5p into endplate chondrocytes [38]. Moreover, we found that miR-31 inhibited the apoptosis of NPCs. This result was in line with a previous study showing that miR-31 regulates cell apoptosis, possibly through the phosphatidylinositol-3 kinase/protein kinase B pathway [11].

Subsequent results of this study revealed that miR-31 bound to NFAT5 3′UTR and inhibited the expression of NFAT5. Consistently, a previous study has underpinned that NFAT5 is a target gene of miR-31 in glioma cells [39]. NFAT5 is known to regulate cell apoptosis [15], which is comparable to our results that NFAT5 overexpression increased apoptosis in NPCs. Increased proinflammatory cytokines were also found in IDD, which is also consistent with a previous study showing that NFAT5 is involved in the inflammatory response [17]. In addition, we found that NFAT5 overexpression decreased ECM synthesis. NFAT5 has been shown to regulate the formation of ECM by controlling the acquisition of collagen through the sonic hedgehog pathway [40]. Another study demonstrated that NFAT5 regulated ECM turnover by regulating the expression of ACAN [41]. These results showed the critical role of NFAT5 in the regulation of ECM. Downstream to NFAT5, we found that reduced NFAT5 expression led to increased expression of *β*-catenin. This result is in line with a previous study showing that NFAT5 inhibited activation of the Wnt pathway [18]. Wnt/*β*-catenin is important in IDD due to their inflammatory functions and roles in regulating cell apoptosis [19, 20].

There are a few limitations to this study. First, the conclusion of this work should be validated in large cohorts due to the relatively small case of patients. Second, although the Wnt/*β*-catenin was implicated in this study, a causal relationship was not established. Further studies should include Wnt/*β*-catenin overexpression or knockdown experiments to study its impact on IDD. Third, we only discerned the interaction between miR-31 and Wnt/*β*-catenin, which requests further research about the specific mechanism between NFAT5 and Wnt/*β*-catenin for validation of the reported signaling axis. Fourth, miR-31 has been documented to target several genes in degenerative NPCs, such as MMP3, SDF-1, CXCR7, and ATF6, thus participating in the IDD progression [13, 38, 42], and we thereby cannot exclude the involvement of these targets in the alleviation of miR-31 in BMSC-EVs in the IDD progression due to the complex microenvironment.

## 5. Conclusion

**Figure 9:**
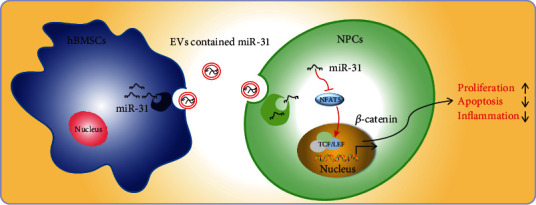
Schematic diagram of the mechanism by which miR-31 in hBMSC-EVs affects IDD. hBMSC-EVs transferred miR-31 to NPCs where miR-31 targets NFAT5 and reduces the expression of NFAT5 and activates the Wnt/*β*-catenin pathway, thus promoting the proliferation of NPCs, inhibiting their apoptosis, and reducing the release of cellular inflammatory factors, ultimately alleviating IDD in mice.

In conclusion, miR-31 from BMSC-EVs can alleviate IDD through inhibition of NFAT5 and activation of the Wnt/*β*-catenin pathway (Figure 9). Therefore, miR-31 and its downstream pathway molecules may be novel therapeutic modalities for IDD treatment that deserves further investigation.

## Data Availability

The datasets generated and analyzed during the current study are available from the corresponding author on reasonable request.

## Abbreviations

IDD: Intervertebral disc degeneration
NP: Nucleus pulposus
NPCs: NP cells
IVD: Intervertebral disc
MSC: Mesenchymal stem cell
EVs: Extracellular vesicles
miR: MicroRNA
NFAT5: Nuclear factor of activated T cells 5
BMSCs: Bone marrow mesenchymal stem cells
ATCC: American Type Culture Collection
3′UTR: 3′untranslated region
ANOVA: Analysis of variance
BSA: Bovine serum albumin
cDNA: Complementary DNA
DMEM: Dulbecco's modified Eagle's medium
EDTA: Ethylenediamine tetraacetic acid
FBS: Fetal bovine serum
FITC: Fluorescein isothiocyanate
KEGG: Kyoto encyclopedia of genes and genomes
MUT: Mutant
oe: Overexpression
NC: Negative control
SDS-PAGE: Sodium dodecyl sulfate-polyacrylamide gel electrophoresis
WT: Wild type
TNF-*α*: Tumor necrosis factor-*α*
HE: Hematoxylin and eosin
DAPI: 4′,6-Diamidino-2-phenylindole
PI: Propidium iodide
TUNEL: Terminal deoxynucleotidyl transferase-mediated dUTP-biotin nick end labeling
CCK-8: Cell counting kit-8
PMSF: Phenylmethylsulphonyl fluoride.

## Supplementary Materials

Supplementary 1 Supplementary Figure 1: hBMSC identification: A: expression of hBMSC surface antigens CD73, TSG1010, and CD105 and non-hBMSC surface antigens CD34, CD45, CD14, CD19CD90, and HLA-DR determined by flow cytometry; B: representative micrographs showing osteogenic, adipogenic, and chondrogenic differentiation of hBMSCs at passage 3; C: Western blot detection of BMSC stemness marker protein Nanog in the hBMSCs at passages 1 and 3. Scale bar = 50 *μ*m. ^∗^*p* < 0.05 vs. the hBMSCs at passage 1. Cell experiments were repeated three times. Supplementary Figure 2: bioinformatics prediction: A: Venn diagram of the predicted downstream genes of miR-31 by the RAID, TargetScan, mirDIP, and miRWalk databases; B: binding sites of miR-31 in the NFAT5 3′UTR predicted by the TargetScan database. Supplementary Figure 3: bioinformatics prediction: A: the related gene network of NFAT5 predicted by GeneMANIA; B: the enrichment of NFAT5 and related pathways analyzed by KOBAS.

Supplementary 2 Supplementary Table 1: primer sequences for qRT-PCR.
