# Supplementary material for: miR-31 from Mesenchymal Stem Cell-Derived Extracellular Vesicles Alleviates Intervertebral Disc Degeneration by Inhibiting NFAT5 and Upregulating the Wnt/β-Catenin Pathway
Source: Stem Cells Int. 2022 Oct 20;2022:2164057. doi: 10.1155/2022/2164057 (PMC9615555; doi:10.1155/2022/2164057)
Supplement: Supplementary 1 — Supplementary Figure 1: hBMSC identification: A: expression of hBMSC surface antigens CD73, TSG1010, and CD105 and non-hBMSC surface antigens CD34, CD45, CD14, CD19CD90, and HLA-DR determined by flow cytometry; B: representative micrographs showing osteogenic, adipogenic, and chondrogenic differentiation of hBMSCs at passage 3; C: Western blot detection of BMSC stemness marker protein Nanog in the hBMSCs at passages 1 and 3. Scale bar = 50 μm. ∗p < 0.05 vs. the hBMSCs at passage 1. Cell experiments were repeated three times. Supplementary Figure 2: bioinformatics prediction: A: Venn diagram of the predicted downstream genes of miR-31 by the RAID, TargetScan, mirDIP, and miRWalk databases; B: binding sites of miR-31 in the NFAT5 3′UTR predicted by the TargetScan database. Supplementary Figure 3: bioinformatics prediction: A: the related gene network of NFAT5 predicted by GeneMANIA; B: the enrichment of NFAT5 and related pathways analyzed by KOBAS. [file 2164057.f1.docx]

**
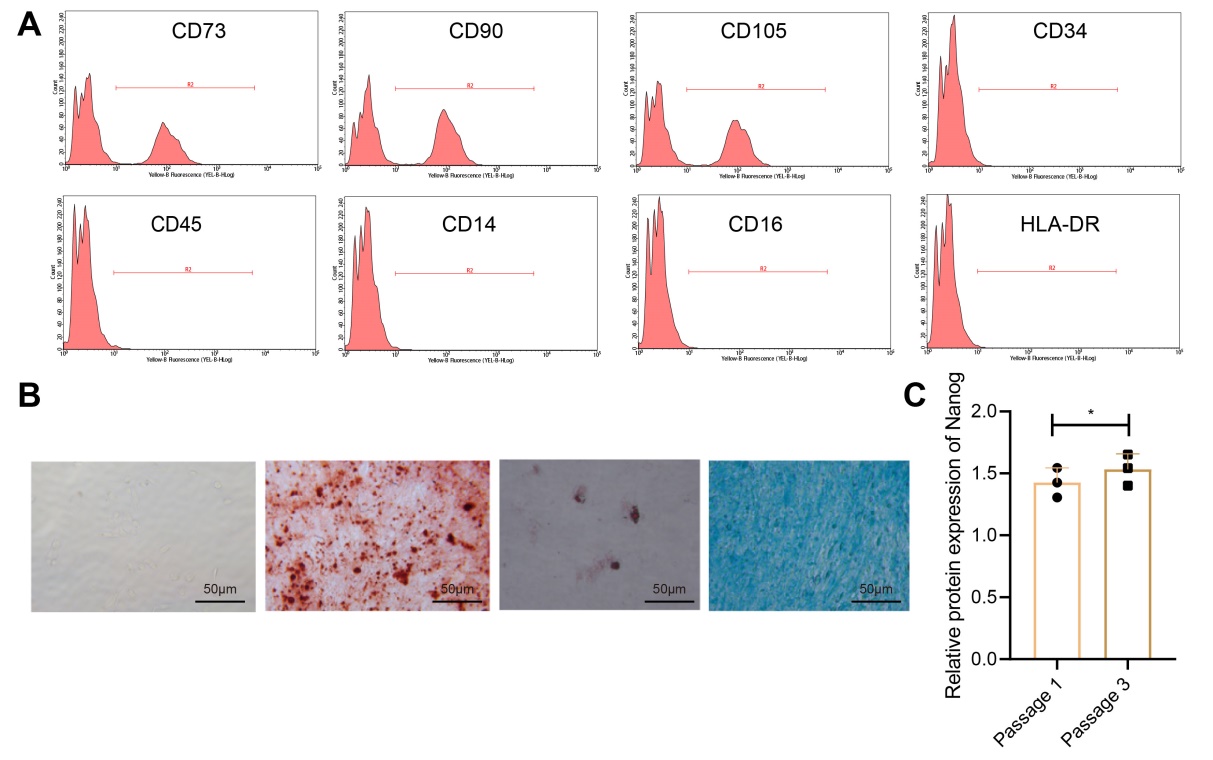
**

**SUPPLEMENTARY FIGURE 1:** hBMSC identification. A: Expression of hBMSC surface antigens CD73, TSG1010, and CD105 and non-hBMSC surface antigens CD34, CD45, CD14, CD19CD90, and HLA-DR determined by flow cytometry. B: Representative micrographs showing osteogenic, adipogenic, and chondrogenic differentiation of hBMSCs at passage 3. C: Western blot detection of BMSC stemness marker protein Nanog in the hBMSCs at passage 1 and 3. Scale bar = 50 μm. **p <* 0.05 vs. the hBMSCs at passage 1. Cell experiments were repeated three times.

**
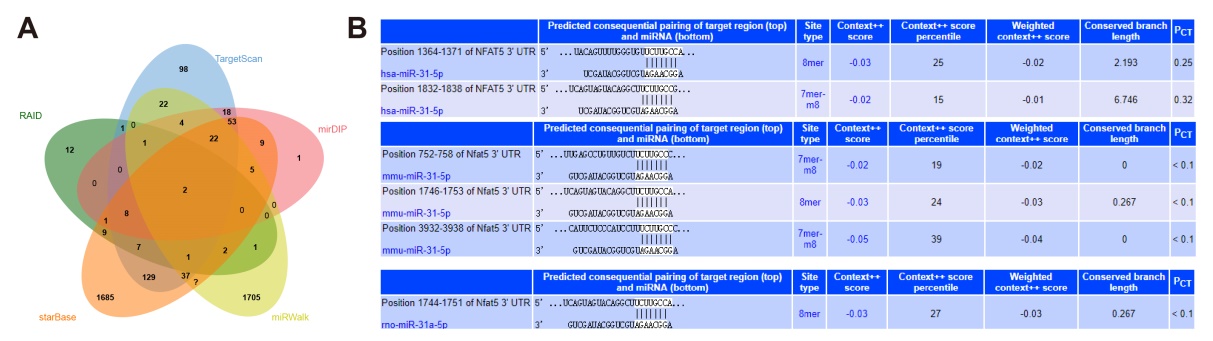
**

**SUPPLEMENTARY FIGURE 2:** Bioinformatics prediction. A: Venn diagram of the predicted downstream genes of miR-31 by the RAID, TargetScan, mirDIP, and miRWalk databases. B: Biding sites of miR-31 in the NFAT5 3’UTR predicted by the TargetScan database.

**
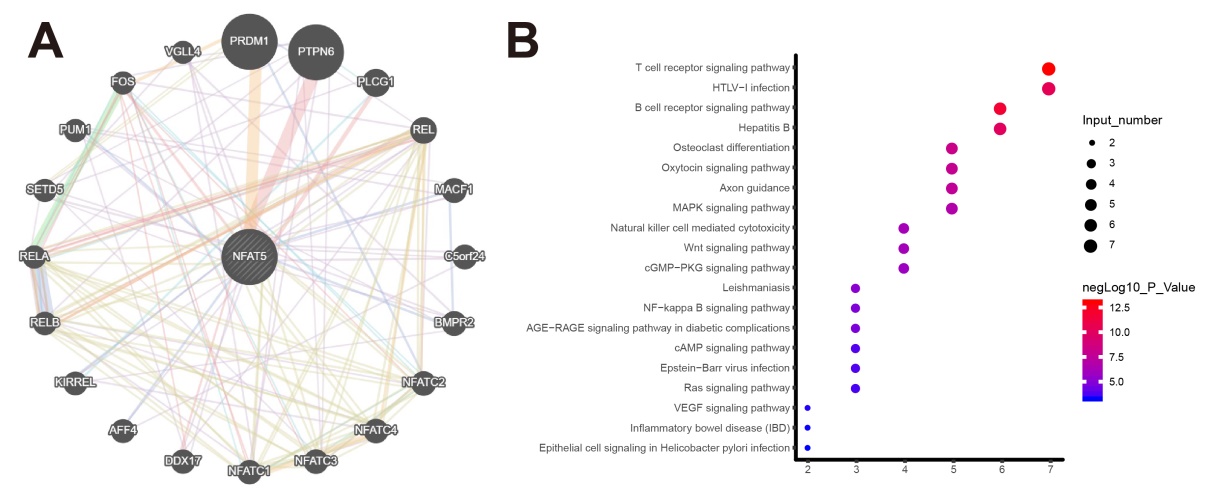
**

**SUPPLEMENTARY FIGURE 3:** Bioinformatics prediction. A: The related gene network of NFAT5 predicted by GeneMANIA. B: The enrichment of NFAT5 and related pathways analyzed by KOBAS.
