# Supplementary material for: miR-31 from Mesenchymal Stem Cell-Derived Extracellular Vesicles Alleviates Intervertebral Disc Degeneration by Inhibiting NFAT5 and Upregulating the Wnt/β-Catenin Pathway
Source: Stem Cells Int. 2022 Oct 20;2022:2164057. doi: 10.1155/2022/2164057 (PMC9615555; doi:10.1155/2022/2164057)
Supplement: Supplementary 2 — Supplementary Table 1: primer sequences for qRT-PCR. [file 2164057.f2.docx]

**Supplementary Table 1**. Primer sequences for qRT-PCR.

| Targets | Primer sequences |
| --- | --- |
| hsa-miR-31 | Forward: 5’-AGGCAAGATGCTGGCATAGC-3’ |
|  | Reverse: Kit universal reverse primer |
| mmu-miR-31 | Forward: 5’-AGGCAAGATGCTGGCATAGCTG-3’ |
|  | Reverse: Kit universal reverse primer |
| cel-miR-39 | Forward: 5’-GGTCACCGGGTGTAAATCAGCTTG-3’  Reverse: Kit universal reverse primer |
| U6 snRNA | Forward: 5’-CTCGCTTCGGCAGCACA-3’ |
|  | Reverse: Kit universal reverse primer |
| Human NFAT5 | Forward: 5’-CCACTCATACCAAGCAGTATG-3' |
|  | Reverse: 5’-CCTGCTGCAATAGTGCATC-3’ |
| Mouse NFAT5 | Forward: 5’-CTCCTCAGATCCAGTTGGTTCA -3' |
|  | Reverse: 5’-GCTGCATGTCTGGTTGGTTTAT -3’ |
| Human β-actin | Forward: 5’-TCACCCACACTGTGCCCATCTACGA-3' |
|  | Reverse: 5’-CAGCGGAACCGCTCATTGCCAATGG-3’ |
| Mouse β-actin | Forward: 5’-GCACTGTGTTGGCATAGAGGTC-3' |
|  | Reverse: 5’-ACGGCCAGGTCATCACTATTGG-3’ |
